# Poly[μ_2_-aqua-[μ_2_-1,1′-(butane-1,4-di­yl)diimidazole]bis­(μ_4_-naphthalene-1,4-dicarboxyl­ato)dimanganese(II)]

**DOI:** 10.1107/S1600536808036787

**Published:** 2008-11-13

**Authors:** Zhi-Qiang Chen, Wen-Zhi Zhang, Qun Xu

**Affiliations:** a College of Chemistry and Chemical Engineering, Qiqihar University, Qiqihar 161006, Heilongjiang Province, People’s Republic of China

## Abstract

In the title compound, [Mn_2_(C_12_H_6_O_4_)_2_(C_10_H_14_N_4_)(H_2_O)]_*n*_ or [Mn_2_(1,4-ndc)_2_(*L*)(H_2_O)]_*n*_, where 1,4-ndc is naphthalene-1,4-dicarboxyl­ate and *L* is 1,1′-(butane-1,4-di­yl)diimidazole, the coordination polyhedron around each Mn^II^ atom is distorted octa­hedral. The water mol­ecule and the *L* ligand are situated across a twofold rotation axis. The Mn^II^ atoms are bridged by 1,4-ndc and *L* ligands, forming a three-dimensional network. O—H⋯O hydrogen bonds are observed within the network.

## Related literature

For general background, see: Ma *et al.* (2003 ▶); Yang *et al.* (2008 ▶).
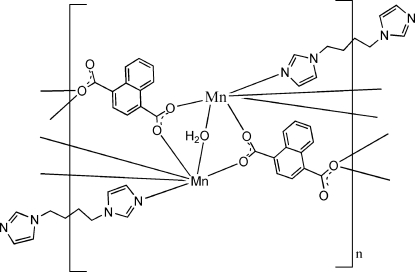


## Experimental

### 

#### Crystal data

[Mn_2_(C_12_H_6_O_4_)_2_(C_10_H_14_N_4_)(H_2_O)]
*M* *_r_* = 746.48
Monoclinic, 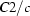

*a* = 18.386 (2) Å
*b* = 14.8887 (18) Å
*c* = 13.9121 (17) Å
β = 126.319 (1)°
*V* = 3068.5 (6) Å^3^
*Z* = 4
Mo *K*α radiation
μ = 0.89 mm^−1^
*T* = 293 (2) K
0.31 × 0.29 × 0.23 mm

#### Data collection

Bruker APEX CCD area-detector diffractometer
Absorption correction: multi-scan (*SADABS*; Bruker, 1998 ▶) *T*_min_ = 0.754, *T*_max_ = 0.814
8475 measured reflections
3032 independent reflections
2643 reflections with *I* > 2σ(*I*)
*R*_int_ = 0.020

#### Refinement

*R*[*F* ^2^ > 2σ(*F* ^2^)] = 0.032
*wR*(*F* ^2^) = 0.081
*S* = 1.06
3032 reflections
226 parameters
H atoms treated by a mixture of independent and constrained refinement
Δρ_max_ = 0.36 e Å^−3^
Δρ_min_ = −0.24 e Å^−3^

### 

Data collection: *SMART* (Bruker, 1998 ▶); cell refinement: *SAINT* (Bruker, 1998 ▶); data reduction: *SAINT*; program(s) used to solve structure: *SHELXS97* (Sheldrick, 2008 ▶); program(s) used to refine structure: *SHELXL97* (Sheldrick, 2008 ▶); molecular graphics: *SHELXTL* (Sheldrick, 2008 ▶); software used to prepare material for publication: *SHELXTL*.

## Supplementary Material

Crystal structure: contains datablocks global, I. DOI: 10.1107/S1600536808036787/ci2711sup1.cif

Structure factors: contains datablocks I. DOI: 10.1107/S1600536808036787/ci2711Isup2.hkl

Additional supplementary materials:  crystallographic information; 3D view; checkCIF report

## Figures and Tables

**Table 1:** Selected bond lengths (Å)

Mn1—N1	2.2157 (17)
Mn1—O1	2.1343 (15)
Mn1—O1*W*	2.2085 (11)
Mn1—O2^i^	2.1535 (14)
Mn1—O4^ii^	2.2115 (14)
Mn1—O4^iii^	2.4148 (13)

Symmetry codes: (i) 
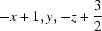
; (ii) 
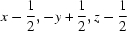
; (iii) 
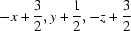
.

**Table 2:** Hydrogen-bond geometry (Å, °)

*D*—H⋯*A*	*D*—H	H⋯*A*	*D*⋯*A*	*D*—H⋯*A*
O1*W*—H1*W*1⋯O3^iii^	0.83 (3)	1.72 (3)	2.5361 (19)	166 (3)

Symmetry code: (iii) 
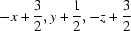
.

**Fig. 1.**
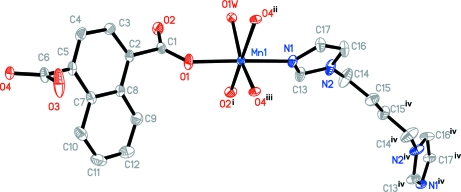
Part of the polymeric structure of the title compound, showing the atomic numbering scheme. Displacement ellipsoids are drawn at the 30% probability level. Symmetry codes: (i) 1-x, y, 3/2-z; (ii) 3/2-x, 1/2+y, 3/1-z; (iii) x-1/2, 1/2-y, z-1/2; (iv) -x, y, 1/2-z.

**Fig. 2.**
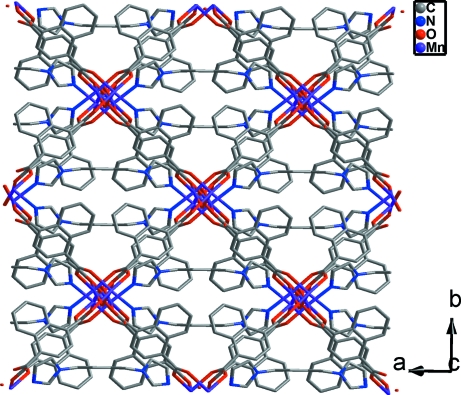
View of the three-dimensional framework of the title compound.

## supplementary crystallographic information

### Comment

Some interesting interpenetrated or entangled metal-organic networks with bis(imidazole)-containing ligands have been documented (Yang *et al.*, 2008). But, flexible ligands such as 1,1'-(1,4-butanediyl)bis(imidazole) (*L*) have not been well explored (Ma *et al.*,  2003). In this work, we used 1,4-naphthalenedicarboxylic acid (1,4-H_2_ndc) and *L* as linkers to obtain a new coordination polymer, [Mn_2_(1,4-ndc)_2_(*L*)(H_2_O)]. We report here its crystal structure.

In the title compound, each Mn^II^ atom displays a distorted octahedral coordination sphere, completed by one N atom from one *L* ligand, four carboxylate O atoms from 1,4-ndc ligand and the O atom of the water molecule (Fig. 1). Both the water molecule and *L* ligand are situated across a twofold rotation axis. Two adjacent Mn^II^ atoms are bridged by carboxylate groups of 1,4-ndc ligands and water molecules forming a three- dimensional network containing [Mn_2_(1,4-ndc)_2_(H_2_O)] units. The network is further strengthened by the coordination of *L* ligands (Fig. 2).

### Experimental

A mixture of 1,4-H_2_ndc (0.5 mmol), *L* (0.5 mmol), NaOH (1 mmol) and MnCl_2_^.^2H_2_O (0.5 mmol) was suspended in 14 ml of deionized water and sealed in a 20-ml Teflon-lined autoclave. Upon heating at 413 K for 3 d, the autoclave was slowly cooled to room temperature. The crystals formed were collected, washed with deionized water and dried.

### Refinement

C–bound H atoms were positioned geometrically (C-H = 0.93–0.97 Å) and refined as riding, with *U*_iso_(H) = 1.2*U*_eq_(C). The water H atom was located in a difference Fourier map and refined freely.

### Crystal data

**Table:**

[Mn_2_(C_12_H_6_O_4_)_2_(C_10_H_14_N_4_)(H_2_O)]	*F*_000_ = 1528
*M**_r_* = 746.48	*D*_x_ = 1.616 Mg m^−^^3^
Monoclinic, *C*2/*c*	Mo *K*α radiation λ = 0.71073 Å
Hall symbol: -C 2yc	Cell parameters from 3032 reflections
*a* = 18.386 (2) Å	θ = 1.1–26.1º
*b* = 14.8887 (18) Å	µ = 0.89 mm^−^^1^
*c* = 13.9121 (17) Å	*T* = 293 (2) K
β = 126.319 (1)º	Block, colourless
*V* = 3068.5 (6) Å^3^	0.31 × 0.29 × 0.23 mm
*Z* = 4	

### Data collection

**Table:**

Bruker APEX CCD area-detector diffractometer	3032 independent reflections
Radiation source: fine-focus sealed tube	2643 reflections with *I* > 2σ(*I*)
Monochromator: graphite	*R*_int_ = 0.020
*T* = 293(2) K	θ_max_ = 26.1º
φ and ω scans	θ_min_ = 1.9º
Absorption correction: multi-scan(SADABS; Bruker, 1998)	*h* = −21→22
*T*_min_ = 0.754, *T*_max_ = 0.814	*k* = −17→18
8475 measured reflections	*l* = −17→17

### Refinement

**Table:**

Refinement on *F*^2^	Secondary atom site location: difference Fourier map
Least-squares matrix: full	Hydrogen site location: inferred from neighbouring sites
*R*[*F*^2^ > 2σ(*F*^2^)] = 0.032	H atoms treated by a mixture of independent and constrained refinement
*wR*(*F*^2^) = 0.081	*w* = 1/[σ^2^(*F*_o_^2^) + (0.039*P*)^2^ + 3.0197*P*] where *P* = (*F*_o_^2^ + 2*F*_c_^2^)/3
*S* = 1.06	(Δ/σ)_max_ = 0.001
3032 reflections	Δρ_max_ = 0.36 e Å^−^^3^
226 parameters	Δρ_min_ = −0.24 e Å^−^^3^
Primary atom site location: structure-invariant direct methods	Extinction correction: none

### Special details

**Table:**

Geometry. All esds (except the esd in the dihedral angle between two l.s. planes) are estimated using the full covariance matrix. The cell esds are taken into account individually in the estimation of esds in distances, angles and torsion angles; correlations between esds in cell parameters are only used when they are defined by crystal symmetry. An approximate (isotropic) treatment of cell esds is used for estimating esds involving l.s. planes.
Refinement. Refinement of F^2^ against ALL reflections. The weighted R-factor wR and goodness of fit S are based on F^2^, conventional R-factors R are based on F, with F set to zero for negative F^2^. The threshold expression of F^2^ > 2sigma(F^2^) is used only for calculating R-factors(gt) etc. and is not relevant to the choice of reflections for refinement. R-factors based on F^2^ are statistically about twice as large as those based on F, and R- factors based on ALL data will be even larger.

### Fractional atomic coordinates and isotropic or equivalent isotropic displacement parameters (Å^2^)

**Table:**

	*x*	*y*	*z*	*U*_iso_*/*U*_eq_	
C1	0.62201 (13)	0.35715 (13)	0.80331 (19)	0.0292 (4)	
C2	0.69094 (14)	0.28719 (14)	0.82714 (18)	0.0284 (4)	
C3	0.78080 (14)	0.30737 (15)	0.90435 (19)	0.0365 (5)	
H3	0.7985	0.3593	0.9502	0.044*	
C4	0.84703 (15)	0.25114 (16)	0.9159 (2)	0.0380 (5)	
H4	0.9076	0.2671	0.9671	0.046*	
C5	0.82279 (14)	0.17325 (14)	0.85222 (18)	0.0312 (5)	
C6	0.89049 (15)	0.12254 (14)	0.84487 (19)	0.0340 (5)	
C7	0.73108 (15)	0.14565 (14)	0.77897 (19)	0.0311 (5)	
C8	0.66391 (14)	0.20353 (14)	0.76583 (18)	0.0287 (4)	
C9	0.57318 (16)	0.17399 (16)	0.6942 (2)	0.0412 (5)	
H9	0.5284	0.2108	0.6844	0.049*	
C10	0.70427 (18)	0.06162 (16)	0.7191 (2)	0.0433 (6)	
H10	0.7475	0.0238	0.7262	0.052*	
C11	0.6163 (2)	0.03575 (17)	0.6515 (3)	0.0547 (7)	
H11	0.5998	−0.0196	0.6132	0.066*	
C12	0.55052 (19)	0.09235 (19)	0.6395 (3)	0.0562 (7)	
H12	0.4905	0.0739	0.5934	0.067*	
C13	0.26433 (15)	0.55518 (18)	0.4740 (2)	0.0458 (6)	
H13	0.2502	0.5058	0.5011	0.055*	
C14	0.12054 (17)	0.6389 (3)	0.3926 (3)	0.0710 (10)	
H14A	0.1202	0.6951	0.4277	0.085*	
H14B	0.1097	0.5909	0.4298	0.085*	
C15	0.04623 (15)	0.6403 (2)	0.2644 (2)	0.0521 (7)	
H15A	0.0518	0.5883	0.2273	0.062*	
H15B	0.0525	0.6934	0.2295	0.062*	
C16	0.25299 (18)	0.68327 (19)	0.3927 (2)	0.0509 (7)	
H16	0.2318	0.7383	0.3541	0.061*	
C17	0.33284 (16)	0.64368 (16)	0.4330 (2)	0.0420 (6)	
H17	0.3763	0.6682	0.4265	0.050*	
N1	0.34027 (11)	0.56325 (13)	0.48419 (16)	0.0334 (4)	
N2	0.21024 (13)	0.62621 (16)	0.42028 (18)	0.0474 (5)	
O1	0.56289 (11)	0.37653 (11)	0.69574 (14)	0.0431 (4)	
O2	0.63083 (10)	0.39108 (10)	0.89143 (13)	0.0384 (4)	
O1W	0.5000	0.54659 (13)	0.7500	0.0258 (4)	
O3	0.88390 (16)	0.13596 (16)	0.75292 (18)	0.0801 (8)	
O4	0.94753 (9)	0.07087 (9)	0.92823 (12)	0.0271 (3)	
Mn1	0.456815 (18)	0.470697 (19)	0.58778 (3)	0.02220 (10)	
H1W1	0.542 (2)	0.578 (2)	0.760 (3)	0.080 (11)*	

### Atomic displacement parameters (Å^2^)

**Table:**

	*U*^11^	*U*^22^	*U*^33^	*U*^12^	*U*^13^	*U*^23^
C1	0.0272 (11)	0.0277 (10)	0.0356 (12)	0.0069 (8)	0.0203 (10)	0.0041 (9)
C2	0.0293 (11)	0.0307 (10)	0.0280 (11)	0.0099 (8)	0.0186 (9)	0.0049 (8)
C3	0.0322 (12)	0.0365 (12)	0.0344 (12)	0.0065 (9)	0.0163 (10)	−0.0075 (9)
C4	0.0250 (11)	0.0478 (13)	0.0333 (12)	0.0089 (10)	0.0129 (10)	−0.0012 (10)
C5	0.0329 (11)	0.0359 (11)	0.0272 (10)	0.0153 (9)	0.0192 (10)	0.0098 (9)
C6	0.0373 (12)	0.0355 (11)	0.0365 (12)	0.0165 (9)	0.0259 (11)	0.0104 (10)
C7	0.0389 (12)	0.0284 (10)	0.0319 (11)	0.0095 (9)	0.0243 (10)	0.0061 (9)
C8	0.0303 (11)	0.0304 (10)	0.0269 (10)	0.0062 (8)	0.0177 (9)	0.0048 (8)
C9	0.0331 (12)	0.0437 (13)	0.0441 (13)	0.0018 (10)	0.0214 (11)	−0.0002 (11)
C10	0.0604 (17)	0.0318 (12)	0.0464 (14)	0.0081 (11)	0.0363 (14)	0.0011 (10)
C11	0.0689 (19)	0.0365 (13)	0.0581 (17)	−0.0120 (13)	0.0373 (16)	−0.0132 (12)
C12	0.0455 (15)	0.0541 (16)	0.0578 (17)	−0.0147 (13)	0.0245 (14)	−0.0098 (14)
C13	0.0282 (12)	0.0585 (15)	0.0465 (14)	0.0064 (11)	0.0197 (11)	0.0183 (12)
C14	0.0301 (14)	0.129 (3)	0.0529 (17)	0.0267 (16)	0.0241 (13)	0.0157 (18)
C15	0.0276 (13)	0.0674 (18)	0.0541 (16)	−0.0057 (12)	0.0204 (12)	−0.0056 (14)
C16	0.0488 (15)	0.0516 (15)	0.0530 (15)	0.0214 (12)	0.0306 (13)	0.0239 (13)
C17	0.0376 (13)	0.0472 (14)	0.0455 (14)	0.0088 (11)	0.0270 (12)	0.0150 (11)
N1	0.0242 (9)	0.0398 (10)	0.0318 (10)	0.0062 (8)	0.0141 (8)	0.0070 (8)
N2	0.0266 (10)	0.0719 (15)	0.0412 (11)	0.0179 (10)	0.0188 (9)	0.0174 (11)
O1	0.0433 (9)	0.0510 (10)	0.0346 (9)	0.0273 (8)	0.0228 (8)	0.0121 (7)
O2	0.0373 (9)	0.0419 (9)	0.0358 (8)	0.0138 (7)	0.0216 (8)	−0.0021 (7)
O1W	0.0245 (11)	0.0252 (10)	0.0319 (11)	0.000	0.0190 (10)	0.000
O3	0.1034 (17)	0.1078 (18)	0.0677 (13)	0.0817 (15)	0.0718 (14)	0.0575 (13)
O4	0.0232 (7)	0.0305 (7)	0.0271 (7)	0.0091 (6)	0.0146 (6)	0.0064 (6)
Mn1	0.01989 (17)	0.02365 (16)	0.02283 (17)	0.00100 (11)	0.01253 (13)	0.00210 (11)

### Geometric parameters (Å, °)

**Table:**

C1—O2	1.245 (2)	C13—H13	0.93
C1—O1	1.256 (2)	C14—N2	1.467 (3)
C1—C2	1.518 (3)	C14—C15	1.470 (4)
C2—C3	1.368 (3)	C14—H14A	0.97
C2—C8	1.423 (3)	C14—H14B	0.97
C3—C4	1.406 (3)	C15—C15^i^	1.497 (4)
C3—H3	0.93	C15—H15A	0.97
C4—C5	1.364 (3)	C15—H15B	0.97
C4—H4	0.93	C16—C17	1.357 (3)
C5—C7	1.420 (3)	C16—N2	1.358 (3)
C5—C6	1.510 (3)	C16—H16	0.93
C6—O3	1.228 (3)	C17—N1	1.358 (3)
C6—O4	1.263 (2)	C17—H17	0.93
C7—C10	1.420 (3)	Mn1—N1	2.2157 (17)
C7—C8	1.426 (3)	Mn1—O1	2.1343 (15)
C8—C9	1.414 (3)	Mn1—O2^ii^	2.1535 (14)
C9—C12	1.362 (4)	Mn1—O1W	2.2085 (11)
C9—H9	0.93	O1W—Mn1^ii^	2.2085 (11)
C10—C11	1.359 (4)	O1W—H1W1	0.83 (3)
C10—H10	0.93	O4—Mn1^iii^	2.2115 (14)
C11—C12	1.401 (4)	O4—Mn1^iv^	2.4148 (13)
C11—H11	0.93	Mn1—O2^ii^	2.1535 (14)
C12—H12	0.93	Mn1—O4^v^	2.2115 (14)
C13—N1	1.323 (3)	Mn1—O4^vi^	2.4148 (13)
C13—N2	1.336 (3)		
			
O2—C1—O1	126.60 (18)	C15—C14—H14B	108.7
O2—C1—C2	117.30 (18)	H14A—C14—H14B	107.6
O1—C1—C2	116.07 (18)	C14—C15—C15^i^	114.7 (3)
C3—C2—C8	119.57 (18)	C14—C15—H15A	108.6
C3—C2—C1	119.00 (19)	C15^i^—C15—H15A	108.6
C8—C2—C1	121.33 (18)	C14—C15—H15B	108.6
C2—C3—C4	121.3 (2)	C15^i^—C15—H15B	108.6
C2—C3—H3	119.3	H15A—C15—H15B	107.6
C4—C3—H3	119.3	C17—C16—N2	106.1 (2)
C5—C4—C3	120.3 (2)	C17—C16—H16	127.0
C5—C4—H4	119.9	N2—C16—H16	127.0
C3—C4—H4	119.9	C16—C17—N1	110.4 (2)
C4—C5—C7	120.31 (18)	C16—C17—H17	124.8
C4—C5—C6	120.2 (2)	N1—C17—H17	124.8
C7—C5—C6	118.88 (19)	C13—N1—C17	104.58 (19)
O3—C6—O4	124.86 (19)	C13—N1—Mn1	124.47 (16)
O3—C6—C5	114.36 (18)	C17—N1—Mn1	130.37 (15)
O4—C6—C5	120.78 (18)	C13—N2—C16	106.84 (19)
C5—C7—C10	121.9 (2)	C13—N2—C14	126.7 (2)
C5—C7—C8	119.17 (19)	C16—N2—C14	126.5 (2)
C10—C7—C8	119.0 (2)	C1—O1—Mn1	140.78 (13)
C9—C8—C2	122.82 (19)	C1—O2—Mn1^ii^	133.19 (13)
C9—C8—C7	118.2 (2)	Mn1—O1W—Mn1^ii^	118.46 (9)
C2—C8—C7	118.94 (19)	Mn1—O1W—H1W1	101 (2)
C12—C9—C8	120.9 (2)	Mn1^ii^—O1W—H1W1	112 (2)
C12—C9—H9	119.5	C6—O4—Mn1^iii^	128.35 (12)
C8—C9—H9	119.5	C6—O4—Mn1^iv^	123.05 (12)
C11—C10—C7	120.9 (2)	Mn1^iii^—O4—Mn1^iv^	106.99 (5)
C11—C10—H10	119.6	O1—Mn1—O2^ii^	89.34 (6)
C7—C10—H10	119.6	O1—Mn1—O1W	89.50 (6)
C10—C11—C12	120.1 (2)	O2^ii^—Mn1—O1W	89.38 (5)
C10—C11—H11	119.9	O1—Mn1—O4^v^	91.01 (6)
C12—C11—H11	119.9	O2^ii^—Mn1—O4^v^	111.20 (5)
C9—C12—C11	120.9 (2)	O1W—Mn1—O4^v^	159.42 (5)
C9—C12—H12	119.5	O1—Mn1—N1	174.19 (6)
C11—C12—H12	119.5	O2^ii^—Mn1—N1	85.26 (6)
N1—C13—N2	112.1 (2)	O1W—Mn1—N1	88.30 (6)
N1—C13—H13	123.9	O4^v^—Mn1—N1	92.93 (6)
N2—C13—H13	123.9	O1—Mn1—O4^vi^	93.37 (6)
N2—C14—C15	114.4 (2)	O2^ii^—Mn1—O4^vi^	174.98 (6)
N2—C14—H14A	108.7	O1W—Mn1—O4^vi^	86.42 (4)
C15—C14—H14A	108.7	O4^v^—Mn1—O4^vi^	73.01 (5)
N2—C14—H14B	108.7	N1—Mn1—O4^vi^	91.86 (6)

Symmetry codes: (i) −*x*, *y*, −*z*+1/2; (ii) −*x*+1, *y*, −*z*+3/2; (iii) *x*+1/2, −*y*+1/2, *z*+1/2; (iv) −*x*+3/2, *y*−1/2, −*z*+3/2; (v) *x*−1/2, −*y*+1/2, *z*−1/2; (vi) −*x*+3/2, *y*+1/2, −*z*+3/2.

### Hydrogen-bond geometry (Å, °)

**Table:**

*D*—H···*A*	*D*—H	H···*A*	*D*···*A*	*D*—H···*A*
O1W—H1W1···O3^vi^	0.83 (3)	1.72 (3)	2.5361 (19)	166 (3)

Symmetry codes: (vi) −*x*+3/2, *y*+1/2, −*z*+3/2.
